# Supplementary material for: Novel Electron-Phonon Relaxation Pathway in Graphite Revealed by Time-Resolved Raman Scattering and Angle-Resolved Photoemission Spectroscopy
Source: Sci Rep. 2017 Jan 19;7:40876. doi: 10.1038/srep40876 (PMC5244369; doi:10.1038/srep40876)
Supplement: Supplementary Information [file srep40876-s1.pdf]

Supplementary: Novel Electron-Phonon Relaxation Pathway in Graphite Revealed by Time-Resolved Raman Scattering and Angle-Resolved Photoemission Spectroscopy

Jhih-An Yang,[1] Stephen Parham,[1] Daniel Dessau,[1,2] and Dmitry Reznik[1]

[1] *Department of Physics, University of Colorado at Boulder, Boulder, CO 80309*

[2] *Center For Experiments on Quantum Materials, University of Colorado, Boulder, CO 80309*

DETAILS OF SIMULATION

Here we discuss the details of the simulation of optical phonon temperature by Eq. 1 in the main text. We used the form $I(t) = (F/2\tau_p) sech^2(t/\tau_p)$ to model the pulse, where F is the absorbed fluence and τ_p is the time duration. We use $\tau_p = 33.15$ fs (FWHM=58.44 fs) so the crosscorrelation width is 90 fs.

The electronic heat capacity can be expressed as:

$$C_{el}(T_{el}) = \frac{\mathrm{d}}{\mathrm{d}T_{el}} \int D(\epsilon) f(\epsilon, T_{el}) \epsilon \mathrm{d}\epsilon, \tag{1}$$

where $D(\epsilon)$ is the density of states for the linear dispersion of bands, and $f(\epsilon, T_{el}) = [\exp(\epsilon/kT_{el}) + 1]^{-1}$ is the Fermi-Dirac distribution by assuming zero chemical potential for simplicity.

The heat capacity for G and K phonons can be expressed as:

$$C_{G/K} = \frac{\mathrm{d}}{\mathrm{d}T} \frac{\hbar\Omega}{\exp(\hbar\Omega/kT) - 1} N, \tag{2}$$

where $\hbar\Omega$ is the phonon energy and N is the number of phonon modes near Γ or K points. For further simplicity we consider G-phonons and K-phonons having the same energy of 200 meV (in reality $\hbar\Omega_G = 200$ meV and $\hbar\Omega_K = 160$ meV).

In our model we used anharmonic decay rate $\tau = 2.4$ ps based on the lifetime of G-phonons measured by our experiment. We also assumed the same decay rate for K-phonons.

We constructed the e-ph decay rate by considering possible transitions. The G-phonons can be emitted only by interband intravalley scattering of electrons due to energy and momentum conservation. Both intraband intervalley and interband intervalley transitions can contribute to the emission of phonons near K points. Note that the phonons from intraband intervalley transitions are not exactly at K points [1]. In general we should integrate all possible states that contribute to the K-phonon emission to obtain the energy transfer rate. Since the main purpose of this model is to explain how K-phonons are responsible for the time delay of G-phonons, the detail of e-ph scattering for K phonons is beyond this scope. In fact how electrons couple to K phonons has a minor influence on the time delay as long as the coupling is strong. Therefore we only considered interband intervalley transitions for K-phonons in our model for simplicity. The exchange rate Γ_{e-ph} for G/K-phonons consequently can be expressed as [2]:

$$\Gamma_{e-ph} = \lambda_{G/K} \left\{ f(\frac{\hbar\Omega}{2}, T_{el}) \left[1 - f(\frac{-\hbar\Omega}{2}, T_{el})\right] (1 + n_{G/K}) - f(\frac{-\hbar\Omega}{2}, T_{el}) \left[1 - f(\frac{\hbar\Omega}{2}, T_{el})\right] n_{G/K} \right\} D(\frac{\hbar\Omega}{2}) D(\frac{-\hbar\Omega}{2}), \tag{3}$$

where $n_{G/K} = [\exp(\hbar\Omega/kT_{G/K}) - 1]^{-1}$ is the G/K-phonon population and $\lambda_{G/K}$ is the e-ph coupling constant.

The energy exchange rate between G and K phonons due to 4-phoonon scattering can be expressed as:

$$\Gamma_{ph-ph}(T_K, T_G) = b[n_K^2(1 + n_G)^2 - (1 + n_K)^2 n_G^2], \tag{4}$$

where b is ph-ph coupling strength.

To solve the coupled differential equations, we used the Euler method [3]. Starting with the initial condition $T_{el,0}$, $T_{G,0}$, and $T_{K,0}$, we take a small time step Δt and evaluate the temperatures $T_{el,1}$, $T_{G,1}$, and $T_{K,1}$ at next time $t_1 = t_0 + \Delta t$. That is,

$$\begin{aligned} T_{el,n+1} &= T_{el,n} + \\ &\Delta t \frac{I(t_n) - \Gamma_{e-ph}(T_{el,n}, T_{G,n}) - \Gamma_{e-ph}(T_{el,n}, T_{K,n})}{C_{el}(T_{el,n})}, \\ T_{G,n+1} &= T_{G,n} + \\ &\Delta t \left(\frac{\Gamma_{e-ph}(T_{el,n} T_{G,n}) + \Gamma_{ph-ph}(T_{K,n}, T_{G,n})}{C_G(T_{G,n})} - \frac{T_{G,n} - T_0}{\tau} \right), \\ T_{K,n+1} &= T_{K,n} + \\ &\Delta t \left(\frac{\Gamma_{e-ph}(T_{el,n}, T_{K,n}) - \Gamma_{ph-ph}(T_{K,n}, T_{G,n})}{C_K(T_{K,n})} - \frac{T_{K,n} - T_0}{\tau} \right) \end{aligned} \tag{5}$$

From the temperatures at t_n, we can evaluate the temperatures at t_{n+1} and so on. The simulated results are convolved with the instrument response that accounts for the finite width of the probe pulse.

The initial condition was set to be room temperature at negative time. The number of phonon modes N in the initial condition is determined by the experimental electronic and phonon temperatures at small positive times. The larger the difference between T_{el} and T_G is, the larger $C_{G/K}$ (and larger N) is. This is because smaller $C_{G/K}$ leads to a larger change in temperature and higher equilibrium temperature the whole system reaches.

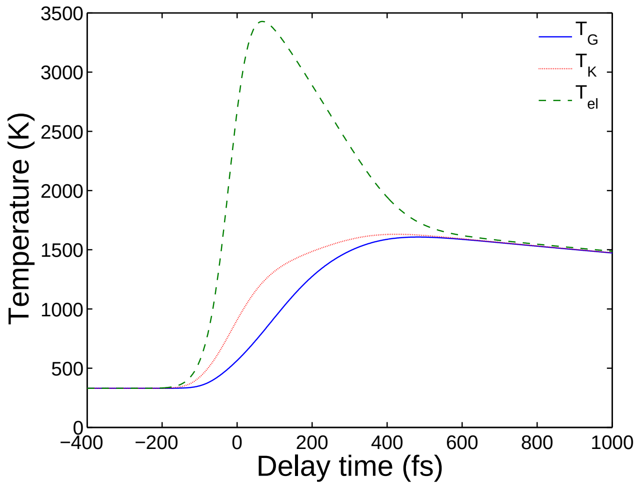

FIG. S1. G-phonon temperature, K-phonon temperature, and electronic temperature as a function of time from the simulation.

F, λ_G, λ_K, and b are adjustable parameters in the simulation to fit experimental results. Here we focus on the region at $t \leq 800$ fs because this is the region where we took most of the Raman data. The long decay of the phonon population has been characterized elsewhere [4]. It can be well described by a single exponential (see the inset of Fig. 2(e) in the main text) due to anharmonic coupling to acoustic phonons. The solution of Eq. 1 in the main text actually follows a single exponential function at long delay time as well. The simulated results are shown in Fig. S1. The parameters for the best fit are $F = 0.0029\ mJ/cm^2$, $\lambda_G = 2.29 \times 10^{-5}\ eV^2m^2/s$, $\lambda_K = 2 \times 10^{-4}\ eV^3m^2/s$, and $b = 2.01 \times 10^{30}\ eV/m^2s$. The parameter F is about 2% of the measured pump fluence 0.15 mJ/cm^2, consistent with 2.3 % for single layer absorption. The χ^2 ($\chi^2 = \sum(T_{exp} - T_{model})^2/\sigma_i^2$, where T_{exp} is the experimental temperature, T_{model} is the simulated temperature, and σ is the errorbar) for the 2T model ($b = 0$) is 76, while the χ^2 for the AHP model ($b \neq 0$) is 33.5. The difference of the χ^2 mainly comes from the deviations from $t = -40$ fs to $t = 300$ fs.

Our model qualitatively describes the role of K-phonons and how they are responsible for the late appearance of G-phonons. We did not consider e-ph scattering for other phonons as their coupling strengths are much less than of G or K phonons.

2D PEAK INTENSITY AND ITS RELATION TO ELECTRONIC TEMPERATURE

The 2D peak intensity on the Stokes side directly measures electronic temperature because it decreases with increasing temperature [5–7] or doping [8, 9] . More electrons in the conduction band enhance the e-e scattering rate and tend to suppress the double resonance responsible for the 2D peak [1, 10, 11]. In our experiment the 2D peak intensity drops quickly around time zero and then recovers more gradually (Fig. S2). Here we used the relation of 2D peak intensity and the electronic linewidth in Ref. [1] since e-e scattering dominates at high temperature [12, 13]. The e-e scattering rate is proportional to temperature [14], so we can rewrite the relation as:

$$I_{2D} = \frac{a'}{\gamma^2} = \frac{a}{T_{el}^2}, \tag{6}$$

where a and a' are constants and γ is the electronic linewidth. We first fit the 2D peak with a Gaussian, whose width was fixed at the energy resolution, plus a linear background (Fig. S2(a) inset). The background should be fit with Planck's law [15], but in a small energy region a straight line is a good fit. Since electrons and G-phonons have the same temperature at t > 500 fs, we found the proportionality a to be 7.42×10^{10} from the intensities at t > 500 fs. We calculated the electronic temperature based on the changes in intensity for

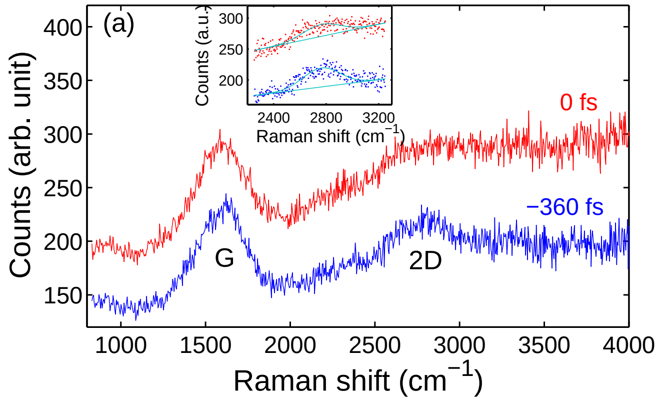

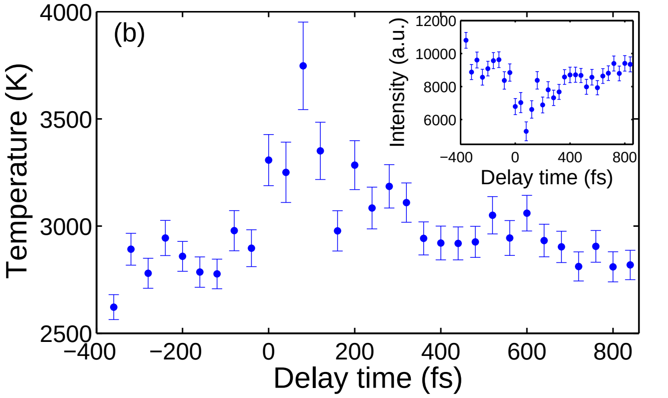

FIG. S2. (a) Stokes Raman spectra at t = -360 fs and t = 0 fs. Inset: Region of the 2D peak with fits to the data (see text). (b) Electronic temperature extracted from Eq. (6). The high electronic temperature at negative times is due to self-pumping by the probe. Inset: Integrated intensity of the 2D peak on the Stokes side as a function of delay time.

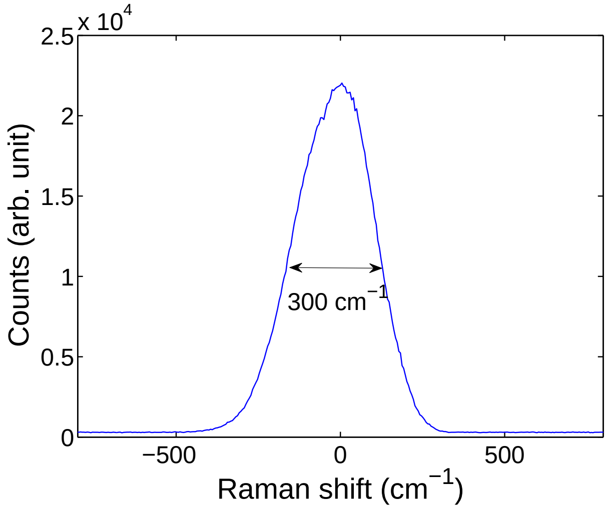

FIG. S3. The energy profile of a probe pulse. The width is about 300 cm^{-1}.

t < 500 fs (Fig. S2(b)). T_{el} extracted from the 2D peak increases rapidly after an optical excitation and reaches 3800 K at t = 80 fs. Compared to trARPES results, the measurement of the 2D peak differs from the real T_{el}. This is because the e-e scattering rate might not follow the simple relation Eq. 6. Self-pumping also causes a deviation from real electronic temperature. As can be seen in Fig. S1, T_e is already over 2000 K at $t = 0$ fs. This explains why T_e at negative times measured from the 2D Raman peak is always above 2500 K, since self-pumping integrates the excitations within about the first 35 fs. This prevents us from extracting real T_e using the 2D peak. Nevertheless, the 2D peak still provides qualitative information about T_e. There is no such a problem for measuring the G phonon population since we did not observe any self-pumping in G phonons.

LASER PROFILE

The frequency profile of a probe pulse is shown in Fig. S3. The full width at half maximum (FWHM) is about 300 cm^{-1}. Now we exam whether the temporal profile is the Fourier-transform-limit. Suppose the laser is described by a Gaussian function in the frequency domain

$$f(E) = e^{-\frac{E^2}{2c^2}} \tag{7}$$

with FWHM= $2\sqrt{2log2}c$. Its Fourier transform is

$$F(t) = \frac{c}{\sqrt{2\pi}\hbar} e^{-\frac{c^2 t^2}{2\hbar^2}}. \tag{8}$$

Therefore the FWHM in the time domain is $2\sqrt{2log2}\hbar/c$. The product of the widths (FWHM) in the time and frequency domains is:

$$\Delta t \Delta E = \left(2\sqrt{2log2}c\right)\left(\frac{2\sqrt{2log2}\hbar}{c}\right) = 8log2\hbar \tag{9}$$

Here $\Delta E = 300\ cm^{-1}$, so we get $\Delta t = 49$ fs. This confirms that our pulse is close to the Fourier limit, given that the measured crosscorrelation width between pump and probe pulses is 90 fs.

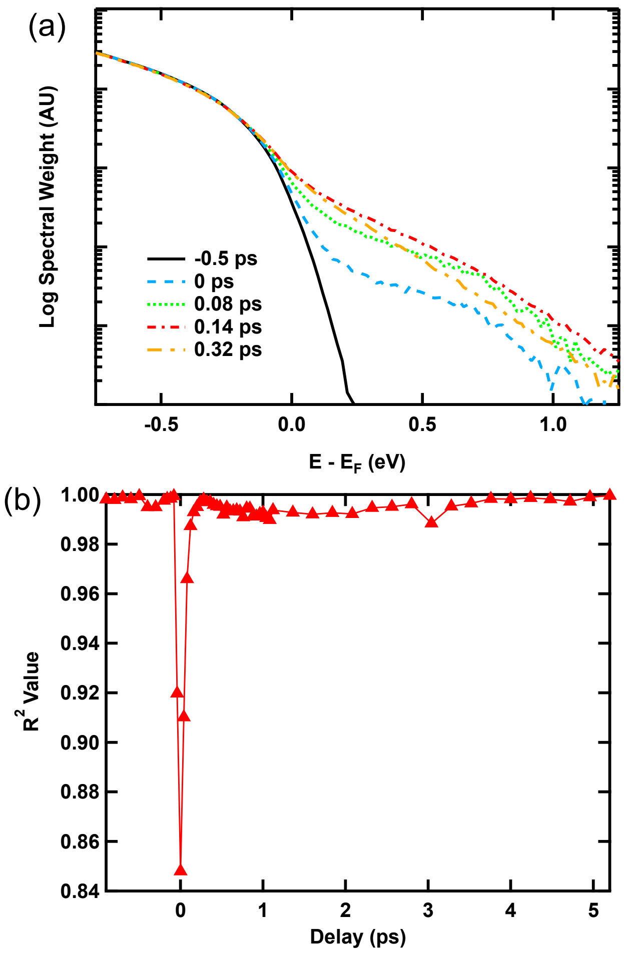

FIG. S4. (a) Momentum-integrated ARPES intensity at early delay times. (b) R^2 value of the fittings at various delay times. (see text)

NONTHERMAL CARRIERS AT EARLY TIMES

Non-thermal behavior of photo-excited carriers is observed in graphite during initial excitation. Fig. S4(a) shows the momentum-integrated ARPES intensity, which is indicative of the occupation function (above E_F). Here we see qualitatively that the $t = 0$ and 0.08 ps traces (gray) do not follow a Fermi-Dirac distribution, which should appear linear above E_F on a log-linear plot.

This is shown quantitatively in Fig. S4(b) as a reduction of the R^2 value of the fitting for time delays near t = 0 (fits are shown in the main text). For $t > 0.12$ ps the system has effectively thermalized, consistent with previous reports [16, 17] and our experimental time resolution of 0.11 ps.

[1] P. Venezuela, M. Lazzeri, and F. Mauri, Phys. Rev. B **84**, 035433 (2011).

[2] P. B. Allen, Phys. Rev. Lett. **59**, 1460 (1987).

[3] J. C. Butcher, *Numerical methods for ordinary differential equations* (John Wiley & Sons, 2008).

[4] H. Yan, D. Song, K. F. Mak, I. Chatzakis, J. Maultzsch, and T. F. Heinz, Phys. Rev. B **80**, 121403 (2009).

[5] P. Tan, Y. Deng, and Q. Zhao, Phys. Rev. B **58**, 5435 (1998).

[6] P. Tan, Y. Deng, Q. Zhao, and W. Cheng, Applied Physics Letters **74**, 1818 (1999).

[7] D. Abdula, T. Ozel, K. Kang, D. G. Cahill, and M. Shim, The Journal of Physical Chemistry C **112**, 20131 (2008).

[8] A. Das, S. Pisana, B. Chakraborty, S. Piscanec, S. K. Saha, U. V. Waghmare, K. S. Novoselov, H. R. Krishnamurthy, A. K. Geim, A. C. Ferrari, and A. K. Sood, Nat Nano **3**, 210 (2008).

[9] A. Das, B. Chakraborty, S. Piscanec, S. Pisana, A. K. Sood, and A. C. Ferrari, Phys. Rev. B **79**, 155417 (2009).

[10] D. M. Basko, S. Piscanec, and A. C. Ferrari, Phys. Rev. B **80**, 165413 (2009).

[11] D. M. Basko, Phys. Rev. B **78**, 125418 (2008).

[12] K. J. Tielrooij, J. C. W. Song, S. A. Jensen, A. Centeno, A. Pesquera, A. Zurutuza Elorza, M. Bonn, L. S. Levitov, and F. H. L. Koppens, Nat Phys **9**, 248 (2013).

[13] In principle the K phonon population should also affect the 2D peak intensity. However under the excitation energy of 3 eV, the momentum of phonons involved in the double resonance process is actually further from K points than those phonons emitted during the electron relaxation, i.e. they are different phonon modes. Since photoexcied electrons thermalize quickly [12], most phonons are emitted from the hot electrons near the Fermi level and therefore have a momentum near K or Γ points. The absence of the 2D peak in the anti-Stokes side also supports that no phonons are generated away from K points, suggesting that the effect of the hot phonon population on the 2D peak is negligible.

[14] M. Schütt, P. M. Ostrovsky, I. V. Gornyi, and A. D. Mirlin, Phys. Rev. B **83**, 155441 (2011).

[15] C. H. Lui, K. F. Mak, J. Shan, and T. F. Heinz, Phys. Rev. Lett. **105**, 127404 (2010).

[16] I. Gierz, F. Calegari, S. Aeschlimann, M. Chávez Cervantes, C. Cacho, R. T. Chapman, E. Springate, S. Link, U. Starke, C. R. Ast, and A. Cavalleri, Phys. Rev. Lett. **115**, 086803 (2015).

[17] I. Gierz, J. C. Petersen, M. Mitrano, C. Cacho, I. C. E. Turcu, E. Springate, A. Stöhr, A. Köhler, U. Starke, and A. Cavalleri, Nat Mater **12**, 1119 (2013).

[18] D. Brida, A. Tomadin, C. Manzoni, Y. J. Kim, A. Lombardo, S. Milana, R. R. Nair, K. S. Novoselov, A. C. Ferrari, G. Cerullo, and M. Polini, Nat Commun **4**, 1987 (2013).
